# A case of POEMS syndrome comprising of a complicated diagnostic procedure: Case report

**DOI:** 10.1097/MD.0000000000037200

**Published:** 2024-03-01

**Authors:** Hai-Ping Huang, Hong-Mei Ran, Zheng-Sheng Li, Juan Xie

**Affiliations:** a Department of Nephrology, The Second Affiliated Hospital of Guizhou University of TCM, Guiyang, China; b Department of Nephrology, Chongqing Hechuan District Hospital of Traditional Chinese Medicine, Chongqing, China.

**Keywords:** misdiagnosis, POEMS syndrome, rare disease

## Abstract

**Rationale:**

This article presents the case of a patient with recurrent chronic diarrhea and cachexia who was misdiagnosed, followed by a literature review to summarize the reasons for misdiagnosis of POEMS syndrome and the treatment strategies.

**Patient concerns:**

The diagnosis and treatment of this patient suggest that with the improvement of M-protein detection levels, the diagnosis of patients with low M-protein levels, such as those with POEMS syndrome, has been greatly aided.

**Diagnoses:**

POEMS syndrome requires polyneuropathy and monoclonal plasma cell proliferation as mandatory diagnostic criteria. Therefore, patients presenting with polyneuropathy should routinely undergo M-protein testing and consider the possibility of POEMS syndrome.

**Interventions:**

The patient, in this case, was treated primarily with relatively conservative immunomodulatory agents.

**Outcomes:**

During follow-up after treatment, the patient’s diarrhea and malnutrition showed significant improvement.

**Lessons subsections:**

POEMS syndrome has low clinical specificity and a high rate of misdiagnosis. However, once a definitive diagnosis is made, the treatment outcome is favorable.

## 1. Introduction

POEMS syndrome (Polyneuropathy, Organomegaly, Endocrinopathy, Monoclonal plasma cell disorder, Skin changes) is a rare etiologic paraneoplastic syndrome associated with plasma cell abnormalities. It was first reported in 1938.^[1]^ In 1980, it was proposed to name this group of syndromes as POEMS syndrome, and the name has been widely employed to date.^[2]^ It is frequently misdiagnosed as an infectious disease and monoclonal gammopathy of uncertain clinical significance. This article provides a review of a case of POEMS syndrome that was not definitively diagnosed by 3 tertiary hospitals in order to enhance understanding of POEMS syndrome.

## 2. Clinical data

### 2.1. Case profile

A 33-year-old female patient was admitted to the hospital with “recurrent edema with anorexia for more than 2 years, exacerbated for 15 days.” Along with malaise, the patient had facial edema and mild edema in both lower limbs without obvious cause more than 2 years ago. The patient had visited a tertiary hospital in Qiandongnan Miao and Dong Autonomous Prefecture, where she was diagnosed with “optic neuritis” and prescribed 13 oral prednisone acetate tablets per day. When fatigue and blurred vision were alleviated, the dosage of prednisone acetate tablets was reduced by 2 tablets per week until the drug was discontinued. After discontinuing the drug, the symptoms of anorexia, fatigue, and gradual weight loss returned, accompanied by facial and lower limb edema. The patient developed persistent diarrhea after consuming “rice tofu” at home over a year ago, with 5 to 10 loose yellow stools per day. The patient subsequently visited a tertiary hospital in Qiandongnan Miao and Dong Autonomous Prefecture, where she was examined, diagnosed with renal insufficiency, hypothyroidism, and treated with symptomatic treatment, such as levothyroxine sodium tablets. However, the symptoms, which included edema, anorexia, fatigue, and diarrhea, persisted. In the subsequent year, the patient lost an excessive amount of weight—approximately 30 kg, and was diagnosed with thrombotic microangiopathy after a renal biopsy at a tertiary hospital in Chongqing more than 2 months ago. The imaging examination revealed splenomegaly, hepatomegaly, and lymphadenopathy, and the axillary lymph node biopsy suggested the diagnosis of lymphatic hyperplasia lesions, which are prone to reactive hyperplasia. Antiplatelet, anticoagulant, and diuretic treatments were administered; the edema improved slightly, whereas anorexia, fatigue, and diarrhea were significantly alleviated. The patient experienced a recurrence of symptoms, including face, abdomen, and lower limb edema 15 days ago, accompanied by a significant increase in anorexia and fatigue. The patient visited a tertiary hospital in Guiyang. Serum immunofixation electrophoresis revealed no obvious abnormalities, and bone marrow aspiration was performed, taking into account poor sampling of bone marrow and thrombocytopenia. After treatments including ascites drainage and blood transfusion, the patient’s symptoms enhanced slightly, and she was transferred to our hospital after being discharged.

### 2.2. Physical examination and auxiliary examination

**Figure 1.**
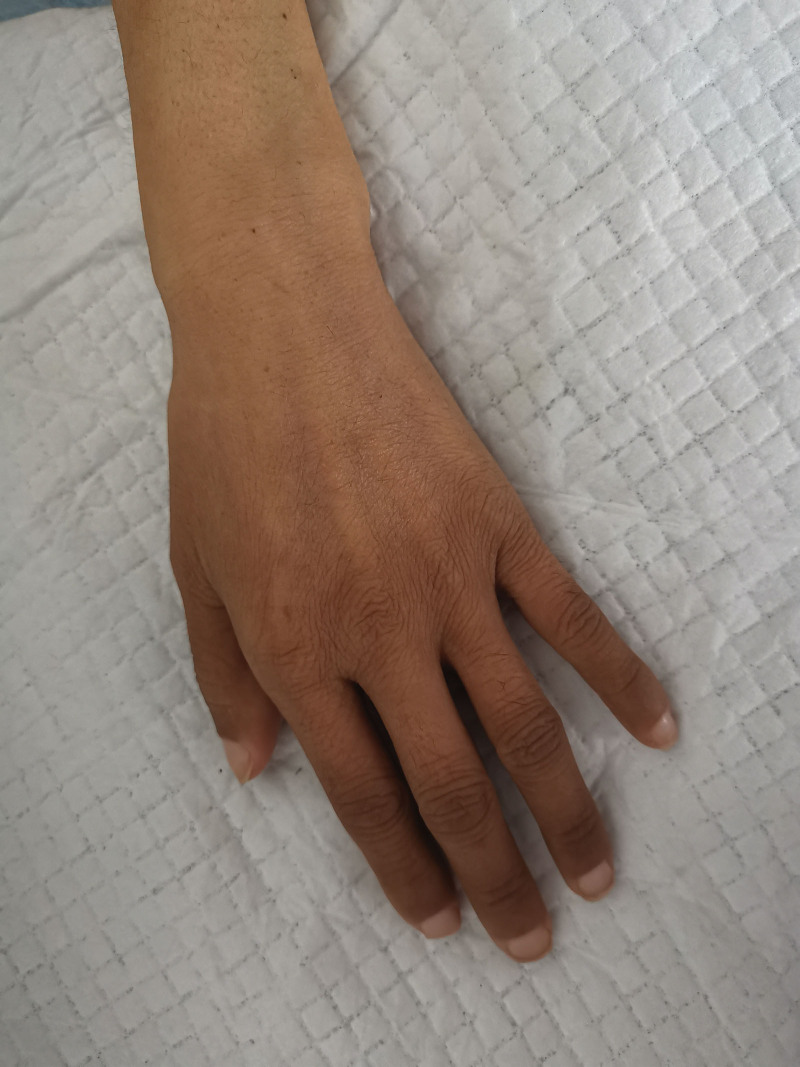
Pigmentation on the skin of distal limbs

**Figure 2.**
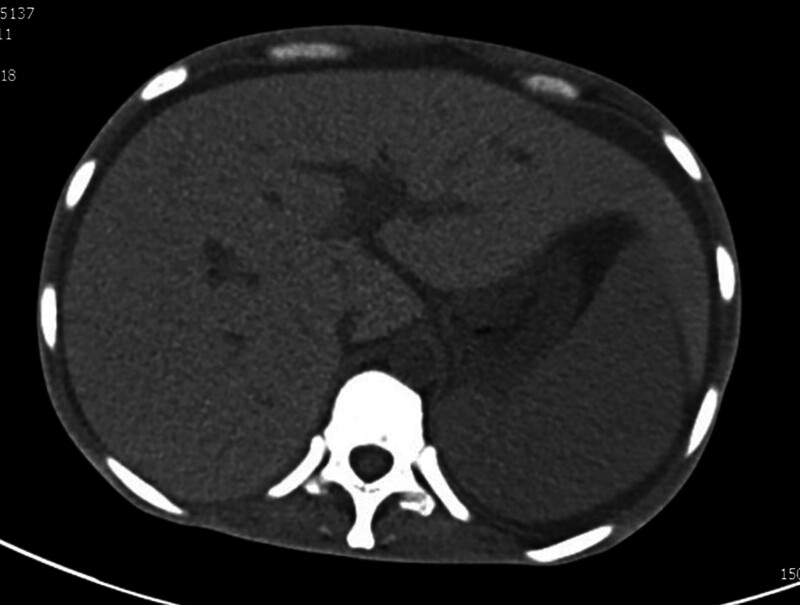
CT revealed hepatosplenomegaly. CT = computed tomography.

**Figure 3.**
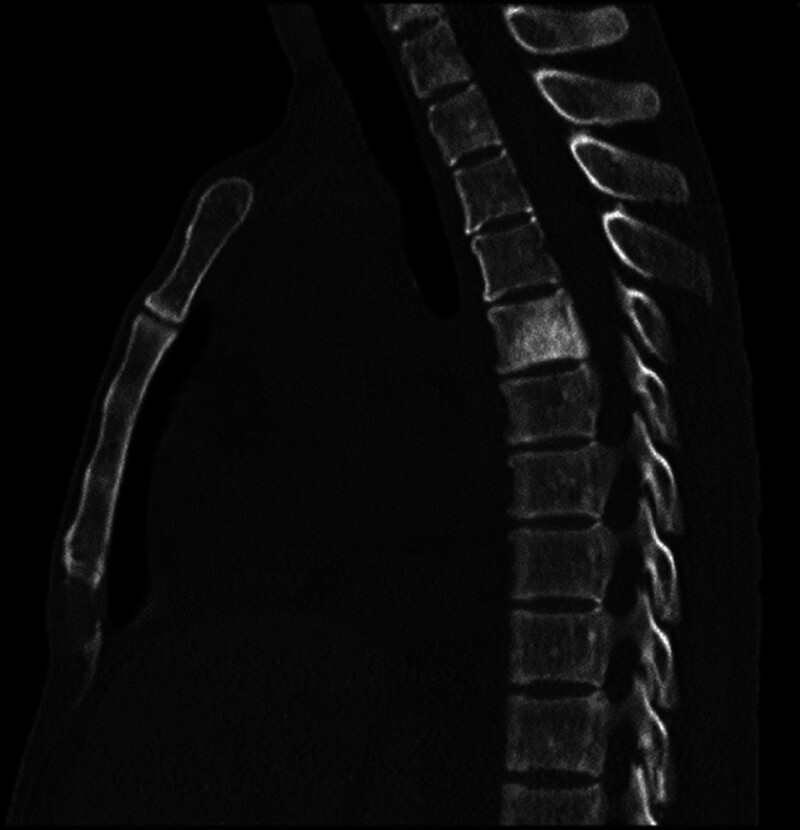
CT revealed the manifestations of bone sclerosis disease. CT = computed tomography.

The patient’s vital signs were stable, but she had poor nutrition and cachexia. The tarsal conjunctiva was slightly pale. Multiple enlarged lymph nodes were palpable bilaterally in the axilla. Multiple lymph nodes were palpable in the central group of the right axillary fossa, and the largest was approximately 2 cm × 2 cm in size. Multiple lymph nodes were palpable in the left axillary fossa, with the largest one measuring approximately 1 cm × 1 cm and was soft, smooth, and without any adhesions. Multiple lymph nodes were palpable bilaterally in the groin, with the largest one measuring approximately 1.5 cm × 1.5 cm and was soft, smooth, and without any adhesions. The remaining lymph nodes could not be felt. The thyroid gland was not enlarged. The thorax was bilaterally symmetric with no deformities. The breath sounds in both lungs were clear, with no rhonchi or rales. The left border of the heart was slightly enlarged. The heart rate was 88 beats per minute. Cardiac rhythm was regular. Auscultatory areas of each valve: the second heart sound in the pulmonary valves area was stronger than the second heart sound in the aortic valves area (P2 > A2), and no pathological murmur was heard in the rest of the auscultation area of each valve. The abdomen as a whole was slightly distended, the abdominal wall was slightly firm, with no tenderness, rebound tenderness, or muscle spasm. The liver edge was palpable 2 cm below the right costal margin and the spleen edge was palpable 3 cm below the left costal margin when the patient inhaled deeply. A fluctuating dreariness was present. The rate of bowel sounds was between 7 and 8 beats per minute. Pigmentation was observed on the skin of both hands (Fig. 1). The bilateral upper extremity muscle strength was grade 4, while the bilateral lower extremity muscle strength was between grade 3 and 4 and muscle tone was normal. There was no induced tendon reflex and vibration sense of the lower extremities decreased. Pathological report of renal biopsy at a different hospital: the total number of glomeruli was 38, no segmental or globular sclerosis, poor capillary loop opening, capillary endothelial hyperplasia and swelling; basement membrane thickening with formation of “double track” sign and absence of obvious lesions in podocytes; mild to moderate mesangial hyperplasia; mild renal tubular lesions. The atrophy area was <25%. There was focal tubular epithelial cell granular degeneration with protein casts and there was focal renal tubular epithelial cell brush margin detachment, with interstitial fibrosis <25%. There was focal and scattered lymphocyte and monocyte immersion, with mild interstitial edema, and swelling of interlobular artery endothelial cells. Myxoid degeneration was occasionally observed in the intima, with narrowing of the lumen and hyaline degeneration of arterioles. Immunofluorescence revealed negative IgA, IgG, IgM, C3, C4c, C1q, κ, λ, fibrinogen, ApoA, AopB, and AopE. Immunohistochemistry revealed negative Pla2R1, CD3, CD20, and CD36, and scattered CD68 (+). Electron microscopy result was not obtained. Blood routine examination after admission: hemoglobin concentration 71 g/L, platelets 91 × 10^9^/L; no fragmented red blood cells were observed. Biochemistry: serum albumin 27.7 g/L, urea 19.18 mmol/L, creatinine 134 μmol/L, calcium 1.69 mmol/L, phosphorus 1.85 mmol/L. Coagulation test: prothrombin duration 36.5 seconds, prothrombin activity 15.5%. Erythrocyte sedimentation rate was 49 mm/h. Anticardiolipin antibodies, 13 ANA antibodies, ANCA, and anti-GBM antibodies were all negative. Urinalysis: WBC + 2 cells/μL ↑, pH 5.00 ↓. Chest and abdominal computed tomography revealed: (1) The patient was emaciated, the mediastinal and axillary lymph nodes were poorly shown, and there was bilateral axillary lymphadenopathy; (2) Splenomegaly and hepatomegaly (Fig. 2), massive fluid in the abdominal cavity and pelvis, and slight thickening of the portal vein; (3) Thickening of the intestinal wall; (4) Calcification in some vertebrae (Fig. 3). Cardiac ultrasound: (1) Mild regurgitation of the mitral and pulmonary valves; (2) Decreased left ventricular diastolic function; (3) Moderate effusion in the pericardial cavity. Immunofixation electrophoresis revealed IgA-λ type. Macrogene testing of ascites did not show DNA virus, *Mycobacterium tuberculosis*, or parasite DNA. Vascular endothelial growth factor (VEGF): 295.65 pg/mL. Electromyography: Indicated multiple sensorimotor nerve damage (predominantly myelin damage, involving axons). Bone marrow aspiration: plasma cells 2.5% and active bone marrow hyperplasia.

### 2.3. Diagnostic process

The patient exhibited cachexia due to severe diarrhea, splenomegaly, hepatomegaly, and lymphadenopathy, along with acral skin pigmentation, hypesthesia, and a mild increase in serum creatinine. The possibility of thrombotic microangiopathy was considered based on the renal biopsy pathology report. A hospital-wide discussion was held on the third day after admission, in which the possible diagnoses of POEMS syndrome, malignancy, abdominal tuberculosis, connective tissue disease, and thrombolytic hemolytic anemia were discussed. Ascites were sent for metagenome testing for further diagnosis and differentiation, serum immunofixation electrophoresis, fragmented red blood cells, autoantibodies, and tumor markers. Serum VEGF was determined. Electromyography and bone marrow aspiration were performed. Finally, a definite diagnosis of POEMS syndrome was made.

The latest diagnostic criteria for POEMS syndrome are those updated by Dispenzieri in 2019^[3]^: (1) Necessary conditions: multiple peripheral neuropathy and monoclonal plasma cell proliferation; (2) Primary conditions: sclerosing bone lesions, Castleman disease, increased serum VEGF; (3) Secondary conditions: organ enlargement (splenomegaly, hepatomegaly or lymphadenopathy), extravascular volume overload (edema, pleural effusion or ascites), endocrine diseases (adrenal glands, thyroid, pituitary, gonads, parathyroid glands, pancreas), skin changes (hyperpigmentation, hirsutism, glomerular hemangioma, hypertrichosis, cyanosis of the hands and feet, flushing, white nails), papilledema, thrombocytosis/polycythemia; Other signs and symptoms: clubbing, weight loss, hyperhidrosis, pulmonary hypertension/restrictive pulmonary disease, thrombogenesis, diarrhea, low vitamin B12. Diagnosis of POEMS syndrome requires 2 prerequisites, at least 1 primary condition, and at least 1 secondary condition. This case met the diagnostic criteria described above.

## 3. Treatment

A literature review was conducted for treating the patient and individualized care was administered.

### 3.1. Symptomatic supportive therapy

A low-sodium diet was prescribed due to edema. Albumin infusion and aggressive diuresis were administered to reduce edema, and an indwelling abdominal drainage catheter was placed to drain ascites. Parenteral nutrition support: energy supply of glucose and fat emulsion was 1:1. Thyroid hormone replacement therapy was prescribed in case of hypothyroid hormone manifestations.

### 3.2. Immunity regulation

Thalidomide and lenalidomide (both thalidomide derivatives) are effective anti-VEGF and anti-cytokine treatments for plasma cell proliferation.^[4]^ Although thalidomide can effectively inhibit VEGF production, it causes toxic neuropathy and must be monitored carefully. Lenalidomide, a second-generation immunosuppressant, is neurotoxic-free and effective against myeloma. It has been shown to be effective when combined with dexamethasone to treat neuropathy and edema, thereby decreasing serum VEGF levels. Lenalidomide and dexamethasone have been shown to enhance neuropathy in 90% of patients diagnosed with POEMS.^[5–7]^

The overall condition of the patient was poor, with cachexia and autonomic disorders. The patient was administered 10 mg of lenalidomide orally for 21 consecutive days. The drug was then discontinued for 7 days. At a 20 mg dosage, dexamethasone was administered to alleviate the patient’s symptoms once a week. The patient must be stable before considering radiotherapy, chemotherapy, or autologous stem cell transplantation.

## 4. Prognosis

The abdominal drainage catheter was removed and the patient was discharged on the 28th day after admission. In the first month following discharge, the patient’s diet had enhanced and she had gained a weight of 2.5 kg. The patient can take care of herself with respect to certain living activities. Renal function and serum albumin were normal, ascites was significantly reduced, and lenalidomide was increased to 15 mg/day. After 6 cycles, dexamethasone was discontinued and lenalidomide 15 mg/day maintenance therapy was used. The patient was advised to undergo additional local radiation therapy, bortezomib therapy, and autologous stem cell transplantation. However, the patient and her family were satisfied with the current improvement of symptoms and were worried about adverse effects and medical expenses. Therefore, the subsequent treatments have not been implemented. The patient is still under follow-up, with normal serum albumin, renal function, and hemoglobin in multiple examinations. The VEGF value decreased gradually (178 pg/mL).

## 5. Discussion

POEMS syndrome is a rare paraneoplastic syndrome with complex clinical manifestations caused by an underlying plasma cell disorder. The pathogenesis is not fully comprehended. The disease is rare and is neglected by clinicians. So far, there were no reports of its occurrence in China, but misdiagnosis and improper treatment of POEMS syndrome are not rare.^[8–10]^

The main reasons for the difficult diagnosis and treatment of this case are as follows: First, the diagnosis did not adhere to the principle of monism, and the subsequent symptoms were ignored. The primary symptoms were emphasized, while the secondary symptoms were disregarded. Local manifestations were disregarded and the systemic symptoms were noticed. Doctors prematurely concluded as they were pleased with the results of a particular laboratory test. In addition, the patient underwent a negative serum immunofixation electrophoresis test at another hospital. Relevant tests were not further conducted, thereby delaying the diagnosis and leading to the wrong outcomes. Since the serum M protein amount in POEMS syndrome is not very high (1–2 g/dL), the positive rate of serum protein electrophoresis is about 75% and about one-quarter of patients with POEMS syndrome are negative for serum M protein, which requires more sensitive serum immunofixation electrophoresis to determine the presence of M protein.^[11]^ Alfonso first reported immunofixation electrophoresis technology in 1964, which included the agar gel protein electrophoresis and immunoprecipitation operations and had the benefits of a short cycle, high sensitivity, and high resolution. Immunofixation electrophoresis can detect M protein with a serum concentration of ≥0.02 g/dL (0.2 g/L) and a urinary concentration of ≥0.04 g/L.^[12]^ Not all patients diagnosed with POEMS are immunofixation electrophoresis-positive, and it is possible that the M protein gradually increases and becomes detectable as the disease progresses. Therefore, a negative serum immunofixation electrophoresis test does not rule out the diagnosis of POEMS syndrome. Regarding this patient, the negative serum immunofixation electrophoresis at another hospital and the positive M protein after admission to our hospital are significant factors in the delayed diagnosis. Even if serum immunofixation electrophoresis does not detect M protein in patients with obvious clinical manifestations, the diagnosis of POEMS syndrome should be considered, particularly in patients with elevated serum VEGF levels. To enhance the M protein detection rate, a serum-free light chain assay with increased sensitivity can be performed.^[13]^ Serum free light chain assay is a sensitive antibody-based assay that detects low concentrations of monoclonal free light chains (κ or λ) in serum. Third, POEMS syndrome is complex and chronically progressive, affecting multiple systems. The gradual onset of clinical symptoms has an impact on the correct diagnosis. In this case, edema was evident, and the patient was promptly diagnosed with renal insufficiency at a different hospital. Renal biopsies demonstrated thrombotic microangiopathy (TMA). TMA is manifested as endothelial cell and subendothelial space edema, with vascular wall thickening and platelet micro-thrombosis, resulting in occlusion of small vessels.^[14]^ However, TMA is a pathological diagnosis term and not a disease. TMA-like changes can manifest in malignant hypertension, systemic lupus erythematosus, scleroderma, antiphospholipid syndrome, and kidney transplant rejection. In a Japanese report, 52 patients diagnosed with POEMS syndrome and renal lesions as described in medical literature, were examined. The most fundamental pathological changes in the kidneys occurred in the glomerulus, manifested as glomerular enlargement, cell proliferation, mesangiolysis, and significant endothelial mesangial cell swelling.^[15]^ POEMS syndrome does not appear to have significant renal pathological manifestations, and it is difficult to confirm the diagnosis of POEMS by renal biopsy, which is another factor contributing to the difficulty in diagnosing the patient. In a case report of POEMS-associated nephropathy, the presence of subendothelial space observed using electron microscopy was mentioned, which may indicate TMA; however, further examination revealed neither thrombosis nor arteriole changes. Therefore, it was likely that microangiopathy is caused by chronic damage to glomerular endothelial cells that was exacerbated during disease outbreaks.^[16]^ In this case, the electron microscopy result was not traced as the patient’s renal biopsy was performed at a different hospital, and a diagnosis of TMA was considered based on the light microscopic observation results of the renal biopsy. If renal pathology demonstrates TMA-like changes, POEMS should be considered based on the patient’s diagnostic history. The cause of this syndrome is unknown, and it is complex. The patient had hepatomegaly, splenomegaly, and lymphadenopathy. Lymph node biopsy was conducted and reactive hyperplasia was considered. Bone marrow biopsy was conducted and no obvious specific lesions were found. Increased serum VEGF is the main feature of the disease.^[3]^ However, the determination of serum VEGF is not a common clinical examination that objectively impacts the correct diagnosis.

Furthermore, few cases in China have been reported, and it is rarely observed in clinical practice. The condition has not gotten enough focus in terms of clinicians’ emotional and rational understanding. The departments within general hospitals are segregated, due to which a comprehensive understanding of the syndrome is lacking. The diagnosis is confirmed by clinicians within the limitations of diseases in their respective specialties, with more one-sided consideration, resulting in the difficult diagnosis of the disease.

There is no standard treatment for POEMS yet. According to a Mayo Clinic retrospective study, radiation therapy can be used as the initial treatment for patients with less bone damage, and has shown favorable therapeutic effects.^[17]^ Only one isolated bone lesion was found in this patient (Fig. 3), and targeted radiotherapy is the most appropriate treatment. The patient and her family, however, were opposed to radiotherapy and refused the treatment. Therefore, we chose to administer systemic chemotherapy. Currently, a VCD chemotherapy regimen (bortezomib + cyclophosphamide + dexamethasone) is the most recommended treatment plan for multiple myeloma. According to Chinese literature, a reduced dose of bortezomib has achieved high efficacy and safety.^[18]^ For patients who are considering a stem cell transplant in the future, it has no impact on future stem cell collection and is superior to lenalidomide in terms of reversal and kidney function protection. The patient had severe diarrhea, which is considered to have been caused by peripheral nerve damage. Thus, this regimen was abandoned as bortezomib would cause peripheral nerve damage and the patient had no intention of undergoing an autologous stem cell transplant due to financial issues. Several studies suggest that lenalidomide-based therapy is also superior in terms of efficacy and safety.^[4–6]^ The patient’s fat content and immunity were extremely low. Thus, for serosal effusion and vascular endothelial cell inflammation, an immunomodulatory RD (lenalidomide + dexamethasone) treatment regimen was selected. The patient’s clinical symptoms have enhanced during the 3-year follow-up period, demonstrating the efficacy of the RD regimen. Thrombosis, decreased platelets, certain hematological toxicity, and neurotoxicity are the adverse effects of lenalidomide. Given the young age of the patient, long-term lenalidomide use should be monitored for adverse effects.

## 6. Conclusion

In conclusion, POEMS syndrome is a rare clonal plasma cell disease with varied clinical manifestations and is difficult to diagnose. Especially given the rarity of patients exhibiting typical symptoms, it is extremely easy to misdiagnose. Clinically, it is important to investigate its pathogenesis, familiarize oneself with its clinical manifestations, comprehend its diagnostic criteria and treatments, enhance one’s understanding of the disease, and reduce the rates of misdiagnosis and missed diagnosis. Early and effective treatment of patients with POEMS syndrome is crucial for enhancing their prognosis.

## Acknowledgments

We are particularly grateful to all the people who have given us help on our article.

## Author contributions

**Conceptualization:** Hong-Mei Ran.

**Data curation:** Hai-Ping Huang, Zheng-Sheng Li, Juan Xie.

**Formal analysis:** Juan Xie.

**Funding acquisition:** Hai-Ping Huang.

**Investigation:** Hong-Mei Ran, Zheng-Sheng Li, Juan Xie.

**Methodology:** Hong-Mei Ran, Juan Xie.

**Project administration:** Hong-Mei Ran, Zheng-Sheng Li.

**Software:** Hai-Ping Huang, Zheng-Sheng Li.

**Supervision:** Zheng-Sheng Li.

**Visualization:** Juan Xie.

**Writing – original draft:** Hai-Ping Huang.

**Writing – review & editing:** Hai-Ping Huang, Hong-Mei Ran, Zheng-Sheng Li, Juan Xie.
